# Comparative Genomics of Two Independently Enriched “*Candidatus* Kuenenia Stuttgartiensis” Anammox Bacteria

**DOI:** 10.3389/fmicb.2012.00307

**Published:** 2012-08-21

**Authors:** Daan R. Speth, Baolan Hu, Niek Bosch, Jan T. Keltjens, Henk G. Stunnenberg, Mike S. M. Jetten

**Affiliations:** ^1^Department of Microbiology, Institute for Water and Wetland Research, Radboud University NijmegenNijmegen, Netherlands; ^2^Department of Environmental Engineering, Zhejiang UniversityHangzhou, China; ^3^Department of Molecular Biology, Nijmegen Centre for Molecular Life Sciences, Radboud University NijmegenNijmegen, Netherlands; ^4^Department of Biotechnology, Delft University of TechnologyDelft, Netherlands

**Keywords:** comparative genomics, anammox, *Kuenenia stuttgartiensis*, metagenomics, enrichment culture

## Abstract

Bacteria capable of anaerobic oxidation of ammonium (anammox) form a deep branching clade within the Planctomycetes. Although the core metabolic pathway of anammox bacteria is largely resolved, many questions still remain. Data mining of the (meta) genomes of anammox bacteria is a powerful method to address these questions or identify targets for further study. The availability of high quality reference data greatly aids such analysis. Currently, only a single “near complete” (∼98%) reference genome of an anammox bacterium is available; that of model organism “*Candidatus* Kuenenia stuttgartiensis.” Here we present a comparative genomic analysis of two “*Ca*. K. stuttgartiensis” anammox bacteria that were independently enriched. The two anammox bacteria used are “*Ca*. K. stuttgartiensis” RU1, which was originally sequenced for the reference genome in 2002 and “*Ca*. K. stuttgartiensis” CH1, independently enriched from a Chinese wastewater treatment plant. The two different “Ca. *Kuenenia*” bacteria have a very high sequence identity (>99% at nucleotide level) over the entire genome, but 31 genomic regions (average size 11 kb) were absent from strain CH1 and 220 kb of sequence was unique to the CH1 assembly. The high sequence homology between these two bacteria indicates that mobile genetic elements are the main source of variation between these geographically widely separated strains. Comparative analysis of the RU1 and CH1 assemblies led to the identification of 49 genes absent from the reference genome. These include a leucyl-tRNA-synthase, the absence of which led to the estimation of the 98% completeness of the reference genome. Finally, a set of 244 genes was present in the reference genome, but absent in the RU1 and CH1 assemblies. These could represent either identical gene duplicates or assembly errors in the published genome. We are confident that this analysis has further improved the most complete available high quality reference genome of an anammox bacterium and will aid further studies on this globally important group of organisms.

## Introduction

Biologically mediated anaerobic ammonium oxidation (anammox) was already predicted in Broda (1977), but first observed as recent as Mulder et al. (1995). Shortly after, the microorganisms responsible for this process were identified as a members of the phylum Planctomycetes (Strous et al., 1999). Since its original discovery, the anammox process has been intensively studied (reviewed in Jetten et al., 2009). The bacteria responsible constitute five related genera that form a deep branching clade in the phylum Planctomycetes (Strous et al., 1999; Schmid et al., 2000, 2003; Kartal et al., 2007; Quan et al., 2008; Jetten et al., 2010). All of these anammox bacteria share a membrane bounded compartment, termed anammoxosome (van Niftrik et al., 2004), where the key reactions in the nitrogen catabolism are thought to occur (van Niftrik et al., 2008). The biochemistry of the core steps in anammox metabolism has been unraveled (Kartal et al., 2011b), yet many questions on these unique organisms still remain to be answered.

Since there is no pure culture or genetic system available for any anammox bacterium, metagenomic analysis of anammox enrichments and natural communities provides the best means to generate new hypotheses and to further our understanding of these organisms. Such metagenomic analysis greatly benefits from the availability of high quality reference data, but for anammox bacteria only a single near complete reference genome (5 contigs; 4.2 Mb; ∼98% complete) is available; that of the model organism “*Candidatus* Kuenenia stuttgartiensis” (hereafter: the Kust genome; Strous et al., 2006). In addition to the Kust genome, a rough dataset containing a draft genome of “*Candidatus* Brocadia fulgida” is published (JGI ID: GM00164; Gori et al., 2011) and, very recently, both a draft genome of “*Candidatus* Scalindua profunda” (JGI ID: 2017108002; van de Vossenberg et al., 2012) and a draft genome of anammox bacterium KSU1 (GenBank ID: NZ_BAFH01000001.1-NZ_BAFH01000004.1; Hira et al., 2012) have become available. These genomes, reflecting the diversity of anammox bacteria, will surely aid in the study of these intriguing organisms. Additionally, the field will benefit from detailed analysis of genomes of closely related species or strains. Until now, no such work has been documented.

Here we report the genomic sequencing of two geographically separated enrichments, containing very closely related (100% at 16S level) anammox bacteria; “*Ca*. K. stuttgartiensis” RU1 and “*Ca*. K. stuttgartiensis” CH1 (hereafter RU1 and CH1 respectively). RU1 is the dominant bacterium in the enrichment culture of which DNA was extracted in 2002 and sequenced for the Kust genome (Schmid et al., 2000; Strous et al., 2006). After 7 years (2002–2009) in continuous culture in Nijmegen, The Netherlands, DNA was re-extracted from this culture and used for resequencing. CH1 is the dominant anammox bacterium in a Chinese wastewater treatment UASB nitrogen removal reactor (Hu et al., 2010). Both bacteria were enriched completely independently, from unrelated inocula and all handling of organisms and DNA was done at different locations (China, France, and The Netherlands), excluding the possibility of cross contamination.

Sequence data of both enrichments were assembled *de novo* and used for comparative analysis with the Kust genome. One of the goals of this comparative analysis was to assess the stability of the reference culture, which indeed could be confirmed. Another goal, determining the degree of variation between the whole genomes of two closely related *Kuenenia* bacteria was also addressed in this study. Furthermore, because of the high sequence identity between the genomes of all three organisms, this analysis provided the opportunity to improve the quality of the Kust reference genome even further.

## Materials and Methods

### Cultures of “*Candidatus* Kuenenia stuttgartiensis”

The enrichment culture of “*Ca*. K. stuttgartiensis” RU1 is the same culture that was originally sequenced for the Kust genome (Strous et al., 2006). It was continuously maintained in reactor systems in our lab (Nijmegen, The Netherlands), with minor modifications resulting in an increased level of enrichment (described in detail in: Strous et al., 1998; Van der Star et al., 2008; Kartal et al., 2011a).

“*Candidatus* Kuenenia stuttgartiensis” CH1 was enriched in a Chinese wastewater treatment plant inoculated with anaerobic digester sludge (Hu et al., 2010).

### Sequencing and assembly

**Figure A1:**
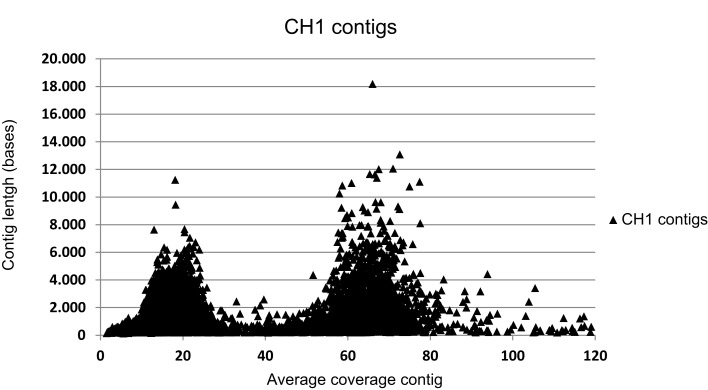
**Correlation of contig coverage and contig length of the 21,343 contigs of the CH1 assembly**. Contigs with an average coverage of 37 or higher were selected for further analysis. The *x*-axis in this graph is truncated for clarity. Due to this truncation of the x-axis 110 contigs with coverage over 120-fold are not shown, but were used in further analysis as part of the “high coverage” group.

DNA was isolated from enrichment cultures of “*Ca*. K. stuttgartiensis” RU1 (Schmid et al., 2000) and “*Ca*. K. stuttgartiensis” CH1 (Hu et al., 2010) and used for Illumina sequencing (GAIIx), yielding 225 (35 bp reads) and 554 Mb (75 bp reads) sequence data, respectively. The obtained reads were assembled *de novo* with the CLC genomics workbench (version 5.1; CLC bio) using default settings. For RU1, this resulted in 2719 contigs averaging 1385 bp. The assembly of CH1 yielded 21,343 contigs averaging 751 bp, which were divided in two clear coverage groups (Figure A1 in Appendix). The high coverage group consisted of 2125 contigs of average length 1840 bases, most of which could be mapped to the Kust genome (Strous et al., 2006). To further improve the assembly of CH1, original reads were extracted from the contigs in the high coverage group and used for a second *de novo* assembly. This assembly yielded 1311 contigs averaging 2906 bp. These assemblies were used for comparative analysis with the Kust genome. RU1 and CH1 assembly data are available at GenBank (Bioproject IDs PRJNA168041 and PRJNA167262 respectively). Comparative analysis was performed using BLAST (McGinnis and Madden, 2004), Mauve (version 2.3.1; Rissman et al., 2009), and the CLC genomics workbench (version 5.1; CLC bio).

### Comparative analysis

To assess the overall similarity between the two assemblies and the reference genome a BLASTn search (with an *E*-value cutoff of 1 × 10^−100^) with both RU1 and CH1 assemblies against the contigs of the Kust genome was performed. Using Mauve, the contigs of both assemblies were ordered with the five contigs of the Kust genome as a template. After ordering, the three draft genomes were aligned using the Progressive Mauve algorithm (Darling et al., 2010). After alignment, the three assemblies were compared using the annotation of the Kust genome. Additionally, the annotated genes of the Kuenenia genome were used as query for a BLASTn search (with an *E*-value cutoff of 1 × 10^−100^) against both the RU1 and CH1 assembly to assess their presence in either assembly. Finally, the reads used for assembly both RU1 and CH1 were mapped on the genes of the Kust genome. The results of these three analyses were merged in the final comparative analysis.

### New “*Candidatus* Kuenenia stuttgartiensis” genes

The alignment using Mauve (described above) revealed approximately 130 kb of sequence on small contigs present (with 95–100% identity) in both new assemblies, which did not align with the Kust genome. These contig sets were iteratively ordered using Mauve, with each other as template. After ordering open reading frames (ORFs) were predicted using Artemis (Carver et al., 2012). These ORFs were used in a BLASTx search against the NCBI nr database. All ORFs without BLAST hit were discarded and overlapping ORFs with a BLAST hit were manually curated. Codon usage of the curated ORFs was determined using codonW (codonw.sourceforge.net; web server: http://bioweb.pasteur.fr/seqanal/interfaces/codonw.html) and compared to the average codon usage of the Kust genome. Expression of the predicted ORFs was assessed using the transcriptome published previously (Kartal et al., 2011b).

## Results

### Comparative analysis of the genome assemblies

The contigs of both assemblies were compared directly to the Kust genome contigs using BLASTn, to determine the level of identity. Of the RU1 contigs, 2616 were on average 99.98 % identical to the Kust genome at the nucleotide level, over a total of 3,623,673 bases, clearly confirming the stability of the enrichment culture. The 0.02% variation that was observed was localized to 45 contigs totaling 26,379 bases (average identity 98.80%), suggesting non-uniform mutation rates across the genome. Surprisingly, 1167 CH1 contigs were on average 99.30% identical to the Kust genome, over a total of 3,176,419 bases. However, in this case the differences were more spread across the genome; on 381 contigs totaling 931,737 bases (average identity 97.85%). Of the RU1 and CH1 assemblies, respectively 103 and 144 contigs had no BLAST hit with the Kust genome (discussed below).

**Figure 1:**
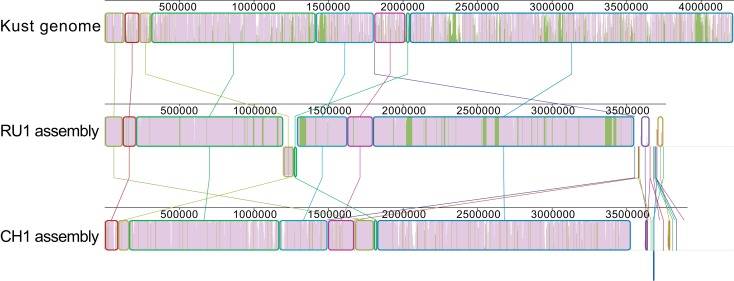
**Alignment of the ordered contigs of the RU1 and CH1 assembly with the Kust genome**. The alignment is built up out of local collinear blocks (colored edges), filled with a similarity plot (purple/green/white). The height of the similarity plot indicates the degree of similarity of the three assemblies at each position. The color of the similarity plot indicates if the sequence is present in all three assemblies (purple), present in the Kust genome and RU1 assembly, but absent from the CH1 assembly (green) or only present in a single assembly (white). Numbers above the similarity plot indicate basepairs.

Additionally, the contigs of both assemblies were prepared for alignment using the “Order contigs” tool of Mauve (Rissman et al., 2009). The ordered assemblies were then aligned using the Progressive Mauve algorithm (Darling et al., 2010) resulting in the alignment shown in Figure 1.

Although the overall synteny of the three assemblies is instantly clear, the alignment also reveals some discrepancies between them. There are many short stretches of the Kust genome to which none of the contigs in either the RU1 or the CH1 assembly aligns. Additionally, visualized in green are 31 genomic regions, up to 44 kb in length and totaling over 300 kb, specifically absent from the CH1 assembly. Finally, it reveals homology between the contigs of the RU1 and CH1 assemblies, which could not be mapped to the Kust genome.

The alignment, in which the annotation of the Kust genome is loaded, was subsequently used to assess the presence of the 4664 Kust genes in either assembly. Additionally, the original reads used for either assembly were mapped to the Kust genome and BLASTn of the Kust genes against the either assembly was performed (Table 1).

**Table 1:** **Number of Kust genes absent from the RU1 and CH1 assemblies analyzed by three independent methods**.

Genes absent from	Mauve	BLASTn (Kust genes vs. contigs)	Unique read mapping
RU1 and CH1	555	740	244
CH1 only	418	471	369
CH1 total (1 + 2)	973	1211	614

*BLASTn was performed with *E*-value cutoff 1 × 10^−100^*.

Combining these three independent analyses confirmed the absence of 359 genes (10 Kust genes to which no reads could be uniquely mapped did produce a BLASTn hit) from the CH1 assembly, which were present in the Kust genome and the RU1 resequencing assembly (Table S1 in Supplementary Material). However, the majority of these genes are annotated as either unknown or (conserved) hypothetical protein, providing little information for a functional comparison between both strains.

On the other hand, mapping of the RU1 resequencing reads resulted in coverage (99.93% average) of all the genes in the Kust genome. However, to 244 Kust genes no reads could be uniquely mapped, explaining their absence from the Mauve alignment and suggesting either gene duplication or errors in the original assembly (Table S2 in Supplementary Material) 77 of these genes were absent from within contigs of the CH1 assembly, while the other 167 marked the sites of breaks between contigs (Table S2 in Supplementary Material).

### New genes of “*Candidatus* Kuenenia stuttgartiensis”

**Figure 2:**
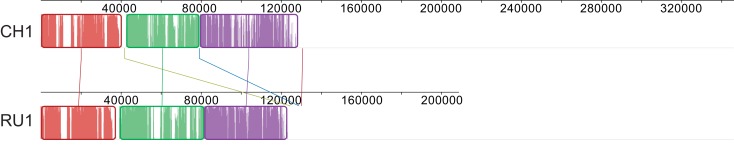
**Alignment of CH1 and RU1 contigs that could not be aligned to the reference genome after iterative ordering**. Alignment is represented as local colinear blocks (colored) filled with a similarity plot. Height of the similarity plot indicates nucleotide identity of both assemblies.

The 103 and 144 contigs from the assemblies of RU1 and CH1 respectively without BLAST hit to the Kust genome could not be ordered by the Mauve “order contigs” algorithm, which requires a reference. However, the alignment does indicate shared sequence between these contigs. To further investigate this we extracted these unordered contigs from the assemblies and iteratively ordered these subsets, using each other as template. This resulted in the alignment shown in Figure 2, indicating that approximately 120 kb of sequence absent from the Kust genome was shared between these two assemblies with very high identity.

Open reading frames of the ordered subsets were predicted and used in a BLASTp search against the NCBI nr database, resulting in 192 and 309 ORFs for RU1 and CH1 respectively. Of these predicted ORFs, 115 were shared between both assemblies, predominantly located in the ordered part indicated in Figure 2 (Table S3 in Supplementary Material). In turn, 60 of these shared ORFs, had >97% homology to genes in the Kust genome and are likely to be sequences from the Kust genome that could not be mapped by the Progressive Mauve algorithm. On the other hand, 50 of these shared ORFs had no high similarity to any species in the NCBI nr database, but codon usage similar to “*Ca*. K. stuttgartiensis.” The high homology of these predicted ORFs in RU1 and CH1, combined with lack of similarity to other known species and similar codon usage to “*Ca*. K. stuttgartiensis” strongly suggests that these ORFs comprise part of the missing 2% of the Kust genome, although their location on the genome cannot be determined from this analysis.

The list of new genes includes a leucyl-tRNA synthetase, the gene which absence led to the 98% completeness estimation of the Kust genome. Additionally, a second (NiFe)-hydrogenase was detected, which was also observed in “*Ca*. S. profunda” (van de Vossenberg et al., 2012). In relation to the core metabolism, two more ammonium transporters and an additional nitrogen regulatory protein P-II were found. To assess the relevance of the predicted new *Kuenenia* gene set, the reads from the published transcriptome (Kartal et al., 2011b) of “*Ca*. K. stuttgartiensis” were mapped on a gene set consisting of the Kust genes and the predicted ORFS of RU1, showing 16 of the new ORFs are highly expressed (RPKM > 100; Table 2). Among the genes with high expression are several unknown and hypothetical proteins, but also the nitrogen regulatory protein P-II and the two ammonium transporters, indicating a role for these in the core metabolism. Furthermore, the hypothetical proteins RU1_045 and RU1_046 could be interesting, because of their high transcription values.

**Table 2:** **Predicted ORFs with high expression in transcriptome analysis**.

Query	Lowest *E*-value	Accession	Description	RPKM
RU1_012c	1.80E−103	YP_003690387	Fis family transcriptional regulator	120.26
RU1_015c	7.60E−97	CAJ74453	Similar to ammonium transport protein amtB possibly fused to histidine kinase	115.68
RU1_016c	2.77E−114	ZP_01386335	Peptide methionine sulfoxide reductase	168.66
RU1_028	2.46E−108	ZP_08829994	Hypothetical protein Rifp1Sym_bw00100	111.62
RU1_033	1.05E−20	YP_004051655	Nitrogen regulatory protein P-II	191.81
RU1_034	4.00E−140	CAJ71754	Ammonium transporter	505.58
RU1_036	5.04E−67	YP_001213278	Hypothetical protein PTH_2728	241.42
RU1_039	7.58E−73	YP_002537074	Flavin reductase domain-containing protein FMN-binding	221.37
RU1_040	9.78E−95	ZP_09413589	Amine acid ABC transporter, permease protein, 3-TM region, His/Glu/Gln/Arg/opine family	103.21
RU1_041	1.98E−69	YP_002250773	ABC-type polar amino acid transport system, ATPase component	202.36
RU1_045c	0	YP_004293396	Glycosyltransferase 36	1,177.89
RU1_046c	3.09E−154	YP_004917811	Unnamed protein product	1,031.84
RU1_090c	7.34E−38	CAJ71211	Strongly similar to *N*-acetylmuramoyl-l-alanine amidase (T7 lysozyme)	851.78
RU1_138c	4.61E−56	YP_463209	Cytoplasmic protein	311.14
RU1_139c	1.29E−82	NP_616688	Cell surface protein	326.24
RU1_173	0	YP_004515866	Leucyl-tRNA synthetase	252.12

**E*-values/accession/description are based on the best BLASTp hit using the amino acid sequence of the predicted ORF as query. RPKM indicates reads mapped, per kilobase of sequence, per million reads mapped on the 4664 Kust genes and the 192 new RU1 genes*.

**Figure 3:**
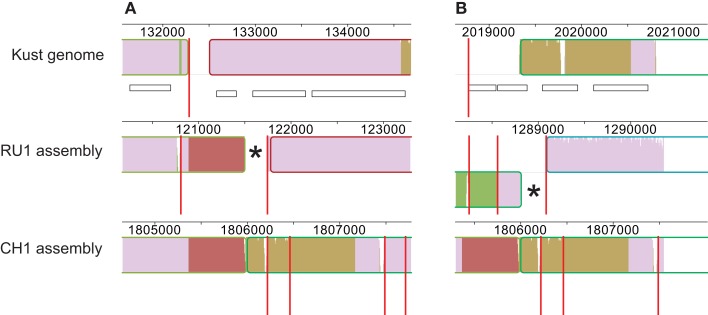
**Contigs in RU1 and CH1 bridging the gap between contigs KustB and KustE of the Kust genome**. The sequence missing from the Kust genome is indicated in red. Red vertical lines mark contig boundaries, white rectangles represent genes, numbers indicate position on the genome. The (*) in both panels marks sequence of the RU1 alignment that is identical to sequence at the start of contig KustE, but not aligned by the Progressive Mauve. **(A)** Alignment of contigs of RU1 (1699, 1254, 256) and CH1 (1429, 701, 1556, 48) with the terminal sequence of contig KustB. **(B)** Alignment of contigs of RU1 (262, 334, 2616) and CH1 (1429, 701, 1556, 48) with the start of contig KustE.

Finally, contig 1254 of the RU1 assembly contained the terminal sequence of contig KustB and the starting sequence of contig KustE of the Kust genome, separated by ∼600 bp. This bridging sequence was also present on contig 1459 of the CH1 assembly, where it also seems bridge the gap between contigs KustB and KustE (Figure 3).

## Discussion

Here we have compared three genome assemblies of “*Ca*. K. stuttgartiensis” anammox bacteria. One of these assemblies, the Kust genome, is the most complete reference genome of an anammox bacterium available to date. The other two are assemblies of two independent enrichment cultures of very closely related anammox bacteria. The resequencing of the enrichment culture used for the Kust genome, termed RU1, was performed to assess culture stability after long-term (7 years) enrichment in our laboratory. The third assembly, termed CH1, was generated to determine the genomic level variation of two fully independently enriched “*Ca*. K. stuttgartiensis” anammox bacteria.

From this study it has become clear that the reference culture maintained in Nijmegen is very stable. The genomic variation between the original sequencing (Strous et al., 2006) and the resequencing performed here is minimal at the nucleotide level (0.02%), and concentrated within a small part of the genome (<1%). However, a significant part of the original assembly (approximately 600 kb) could not be accounted for in the resequencing. This can be partially explained by the occurrence of duplicate genes in the genome, since the sequencing and assembly methods have limited capabilities to discriminate between highly similar duplicates. Read mapping indeed revealed a set of 244 genes to which no reads of the resequencing (or the CH1 sequencing) could be uniquely mapped. These 244 genes could be actual duplicates present in the genomes, or errors in the Kust genome. The latter is relatively likely for a subset of 77 of these genes, for which the upstream and downstream sequences were continuous on a contig of the CH1 assembly (Table S2 in Supplementary Material). However, it should be noted that such “genes falling out of contigs” was not observed for the resequencing, thus could also be explained by mobile genetic elements.

Indeed, most of the observed variation between the two enrichments sequenced seemed to originate from mobile genetic elements. Although the overall sequence identity between the CH1 assembly and the Kust genome is higher than 99% on 3.1 million bases, more than 300 kb containing 369 genes are specifically absent from CH1. These sequences were not present in the low coverage contigs that were discarded (data not shown) and not detectable in three independent analyses, thus appear to be genuinely absent from the CH1 genome. The majority of these 369 missing genes is annotated as hypothetical or unknown protein, hampering the drawing of conclusions from these differences. On the other hand, this set of genes not present in CH1 clearly sets it apart from the reference culture maintained in Nijmegen, despite its extremely high sequence conservation. Perhaps the long doubling time of anammox bacteria slows proliferation of mutations throughout the population, setting the stage for a more dominant role of mobile genetic elements. This is consistent with the large number of transposases found in the Kust genome.

The presence of a second (NiFe)-hydrogenase in “*Ca*. K. stuttgartiensis” is consistent with the recent genome of “*Ca* S. profunda” in which two hydrogenases are also present (van de Vossenberg et al., 2012). The two amtB type ammonium transporters further add to the already high redundancy in the Kust genome (Strous et al., 2006). Their physiological relevance will remain to be shown, but the transcription levels suggests a prominent role for RU1_034 in the ammonium metabolism. As shown in Table 2, some of the new genes are highly expressed, most notably RU1_045, encoding a glycosyltransferase 36 like protein and RU1_046, encoding an unknown protein. Their transcription level places them amongst the 200 highest expressed genes in “*Ca*. K. stuttgartiensis.” However, to determine the role of these predicted genes within the cell biology of *Kuenenia* will require further biochemical studies.

## Conclusion

We have presented a comparative analysis of two very closely related “*Ca*. K. stuttgartiensis” bacteria. This analysis has shown that mobile genetic elements are a major source of variation in these slow growing bacteria. Furthermore, our analysis has revealed 49 new genes of “*Ca*. K. stuttgartiensis” and allowed closing one of the five gaps in the Kust genome. We hope that this analysis will be beneficial to further study of these intriguing organisms.

## Conflict of Interest Statement

The authors declare that the research was conducted in the absence of any commercial or financial relationships that could be construed as a potential conflict of interest.

## Supplementary Material

The Supplementary Material for this article can be found online at http://www.frontiersin.org/Evolutionary/and/Genomic/Microbiology/10.3389/fmicb.2012.00307/abstract

Supplementary Table S1 **Genes specifically absent from CH1**.

Supplementary Table S2 **Kust genes without unique reads**. Yellow background indicates genes of which the up and downstream sequence is continuous in the CH1 assembly.

Supplementary Table S3 **Predicted ORFs not aligning to the Kust genome**. Yellow background indicates best hit to the Kust genome. Data results from a BLASTp search of the predicted ORFS against the NCBI nr database.
